# Evaluating the Accuracy of the SIL Score for Predicting the Sepsis Mortality in Emergency Department Triages: A Comparative Analysis with NEWS and SOFA

**DOI:** 10.3390/jcm13247787

**Published:** 2024-12-20

**Authors:** German Devia Jaramillo, Lilia Erazo Guerrero, Natalia Florez Zuñiga, Ronal Mauricio Martin Cuesta

**Affiliations:** 1 Department of Emergency Medicine, Hospital Universitario Fundación Santafé de Bogotá, Bogotá 110111, Colombia; lilia.erazo@fsfb.org.co (L.E.G.); nataliaflzu@gmail.com (N.F.Z.); 2 School of Medicine and Health Sciences, Universidad del Rosario, Bogotá 111221, Colombia; ronal.martin@urosario.edu.co

**Keywords:** NEWS, SOFA, sepsis, diagnosis, mortality

## Abstract

**Background/Objective:** Sepsis is a disease with a high mortality rate, which emphasizes the importance of developing tools for the early identification of high-risk patients and to initiate timely treatments to reduce mortality. The SIL score is a scale that uses the shock index and arterial lactate level to identify early on the patients that are at a high risk of in-hospital mortality due to sepsis. The purpose of this study was to validate the SIL score as a tool for estimating the probability of sepsis in-hospital mortality from the triage room in emergency departments. Additionally, the advantages of the SIL score were evaluated in comparison with NEWS and SOFA. **Methods:** All of the patients with suspected sepsis were prospectively recruited from the triage room in an emergency department. The SIL score, as well as other evaluation scales, were calculated for these patients. The sensitivity, specificity, predictive values, and areas under the curve (AUC) of each scale were assessed to predict mortality. **Results:** This study included 315 patients. The total mortality of the cohort was 20.4%. Of the total population, 35.5% were in septic shock. The SIL, NEWS, and SOFA scores had similar sensitivities, approximately 60%; however, a higher specificity was documented in the SIL score over the other scales (67%). The SIL score demonstrated superior discriminatory ability compared to the NEWS and SOFA scores (AUC = 0.754, *p* = 0.01). **Conclusions:** The SIL score proved to be a useful tool for predicting in-hospital mortality due to sepsis. Its discriminatory ability surpasses that of other evaluated scales. Therefore, the SIL score can be successfully implemented in the triage room of emergency departments to improve the identification and early management of patients with sepsis.

## 1. Introduction

According to the WHO, in 2017, approximately 48.9 million cases and 11 million sepsis-related deaths were reported worldwide, accounting for nearly 20% of all global deaths [1]. Approximately 85% of these deaths occurred in low- and middle-income countries [1]. The delay in promptly addressing sepsis diagnoses in emergency rooms has been correlated with reduced administration times for interventions, such as initiating antibiotics, which subsequently leads to increased mortality [2,3]. Given the high global mortality rates, particularly in low- and middle-income countries, it is essential to develop rapid and accurate predictive models of the mortality in sepsis that can be applied during triage. Hence, it is imperative to have tools for the early recognition of sepsis to ensure prompt treatment, thereby enhancing patient prognoses. Despite advancements in treatments, there has been no resuscitation protocol shown to have reduced mortality since 2001 [4]. For this reason, while there may not be “ideal” protocols for reducing mortality, it follows that the earlier a patient with a high probability of sepsis-related death is identified, the more likely they are to receive timely standard therapies, thus ultimately improving their prognosis.

At present, there is no universally recommended scale for predicting sepsis-related mortality in emergency department triages. While commonly utilized, both the Quick Sequential Organ Failure Assessment (qSOFA) and the National Early Warning Score (NEWS) have been subjected to controversy regarding their predictive accuracy [5].

This highlights the necessity for novel prediction tools, especially in triage, to prevent delays in care, thereby contributing to more timely forewarning in order to reduce patient mortality. The Shock Index/Lactate (SIL) score was previously developed by our team using the relationship between a clinical variable and one that reflects the patient’s metabolic behavior. We think that, through using a hemodynamic variable added to a metabolic variable, the scale could be closer to the physiology of sepsis and therefore offer better prognostic performance than scales that only use clinical variables. This is why we established that the relationship between the shock index and the lactate value be determined upon entry to the triage room. Therefore, this study aimed to validate the SIL score as a tool for identifying the probability of sepsis in-hospital mortality in the triage room of emergency departments and to assess its competitive advantages compared to other scales.

## 2. Material and Methods

### 2.1. Study Design

This article is a prospective diagnostic test validation study that was aimed at determining the predictive capability of in-hospital mortality using the SIL score and comparing it with other prediction scales such as qSOFA, NEWS, and Sequential Organ Failure Assessment (SOFA).

### 2.2. Eligibility Criteria

This study included patients over 18 years old with suspected sepsis (according to the Sepsis-3 consensus) [6] treated at a high-complexity care institution. All of the patients who met the eligibility criteria were sequentially included until the planned sample size was reached. Patients who had been referred with previous treatment at another institution and pregnant patients were excluded. We excluded pregnant patients because the prediction scales have not been validated in this population. Likewise, we excluded referred patients because it would not be possible to know their final outcome.

The patients were seen by a nurse in the triage area of an emergency department, and each nurse received specific training for identifying the patients with suspected infection. Once the nurse identified a patient with symptoms of infection (fever, cough, dysuria, diarrhea, etc.), the NEWS score was calculated. If the score was ≥5 points, the patient was admitted to the emergency room, where the emergency physician calculated the qSOFA and NEWS scales, as well as the SIL score, and they then assessed whether the patient had clinical signs of sepsis. If positive, tests were requested to calculate the SOFA score, and management was then initiated according to institutional guidelines. All of the patients included in this study were followed throughout their hospital stay until the confirmation of sepsis, and this was determined through culture reports and/or diagnostic imaging, all of which were based on the Sepsis-3 guidelines [6].

### 2.3. SIL Score

The SIL score is a tool designed for use in the triage area, and it is aimed at identifying those patients with suspected sepsis who have a high probability of mortality. This score consists of the relationship between the value of the shock index and the lactate level, which are determined upon admission to the emergency department. Its derivation and initial validation were conducted in a population of septic patients at a high-complexity institution in Bogotá. The results of this work were presented at the research contest of the III International Congress of Emergency and Emergencies, ACEM—Universidad de Antioquia, Medellín, on 20th and 21st October, 2017 (unpublished information).

The SIL score integrates the lactate level and shock index, combining clinical aspects, predominantly hemodynamic, with cellular metabolic aspects. The shock index (heart rate/systolic pressure) [7] serves as a clinical variable for a quick application that reflects the hemodynamic status, while the arterial lactate level is a paraclinical variable that indicates metabolic alterations.

The score ranges from 0 to 6 points, and its ability to estimate mortality is directly proportional to the score’s value (Table 1).

### 2.4. Sample Size

The pROC package of R Version 1.4.1106 © 2009-2021 RStudio, PBC was utilized for sample size calculation. A disease prevalence of 18% was estimated, with a significance level of 95% and a power of 80%. A difference of 0.1 between the areas under the curve (delta) was deemed relevant. Based on these parameters, a minimum sample size of 232 patients was calculated.

### 2.5. Statistical Analysis

The information recorded in the REDcap data collection tool was reviewed for inconsistencies or duplications. A descriptive analysis of the variables under study was conducted using frequency distribution for categorical variables. For continuous variables, measures of the central tendency and dispersion were calculated employing the mean and standard deviation or median and interquartile range (IQR) depending on the data distribution (normal or non-normal), and the Shapiro–Wilk normality test was also applied. The sensitivity, specificity, and the area under the curve (AUC) were determined using standard formulas for a binomial proportion along with 95% confidence intervals. Positive predictive values (PPVs) and negative predictive values (NPVs) were adjusted according to the prevalence of the disease found in this study. Additionally, the association between the categorical variables and mortality was explored by calculating the relative risk and by conducting independence tests using the chi-square statistic. A *p*-value that was less than 0.05 was considered statistically significant. All of these statistical calculations were performed using R Version 1.4.1106 © 2009-2021 RStudio, PBC.

## 3. Results

**Figure 1:**
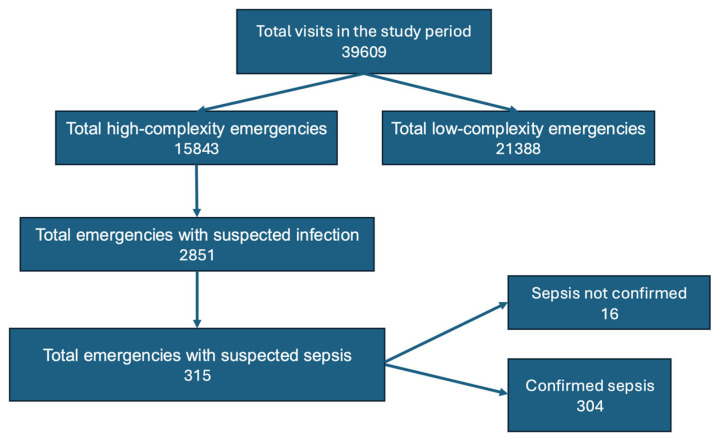
Flowchart for patient inclusion.

A sample of 299 subjects was used for the analyses, and these were selected from the total number of patients. This sample was collected from June 2023 to March 2024 (Figure 1).

Of the 315 patients with suspected sepsis, the diagnosis was confirmed in 94.9% (*n* = 299). Among them, 124 (41.5%) were male with a median age of 72 years (IQR 23-104). The mortality of the cohort was 20.4%, and the mortality due to septic shock was 39.6%. The only comorbidity significantly associated with mortality was chronic renal failure OR 4.11 (95% CI: 1.84–9.08) (Table 1).

The diagnosis of sepsis alone was documented in 193 (64.5%) patients with a mortality rate of 9.8% (OR 0.16, CI: 0.08–0.30; *p* < 0.001). Shock sepsis diagnosis was made in 106 (35.5%) patients with a mortality rate of 39.6% (OR 5.94; 95% CI: 3.25–11.21, *p* < 0.001) (Table 2).

**Figure 2:**
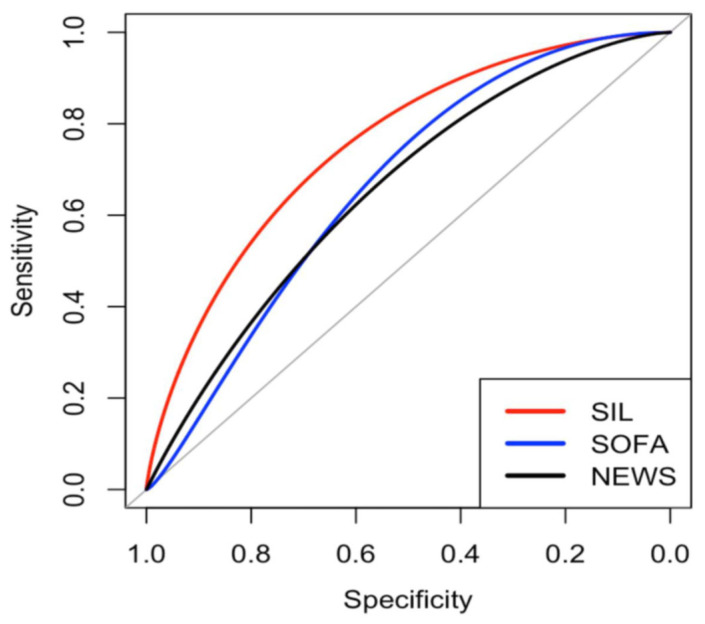
Comparison of the prediction tools’ receiver operating characteristic curves. AUC SIL: 0.754 (95% CI: 0.680–0.825), AUC SOFA: 0.663 (95% CI: 0.578–0.750), and AUC NEWS: 0.655 (95% CI: 0.573–0.733).

**Figure 3:**
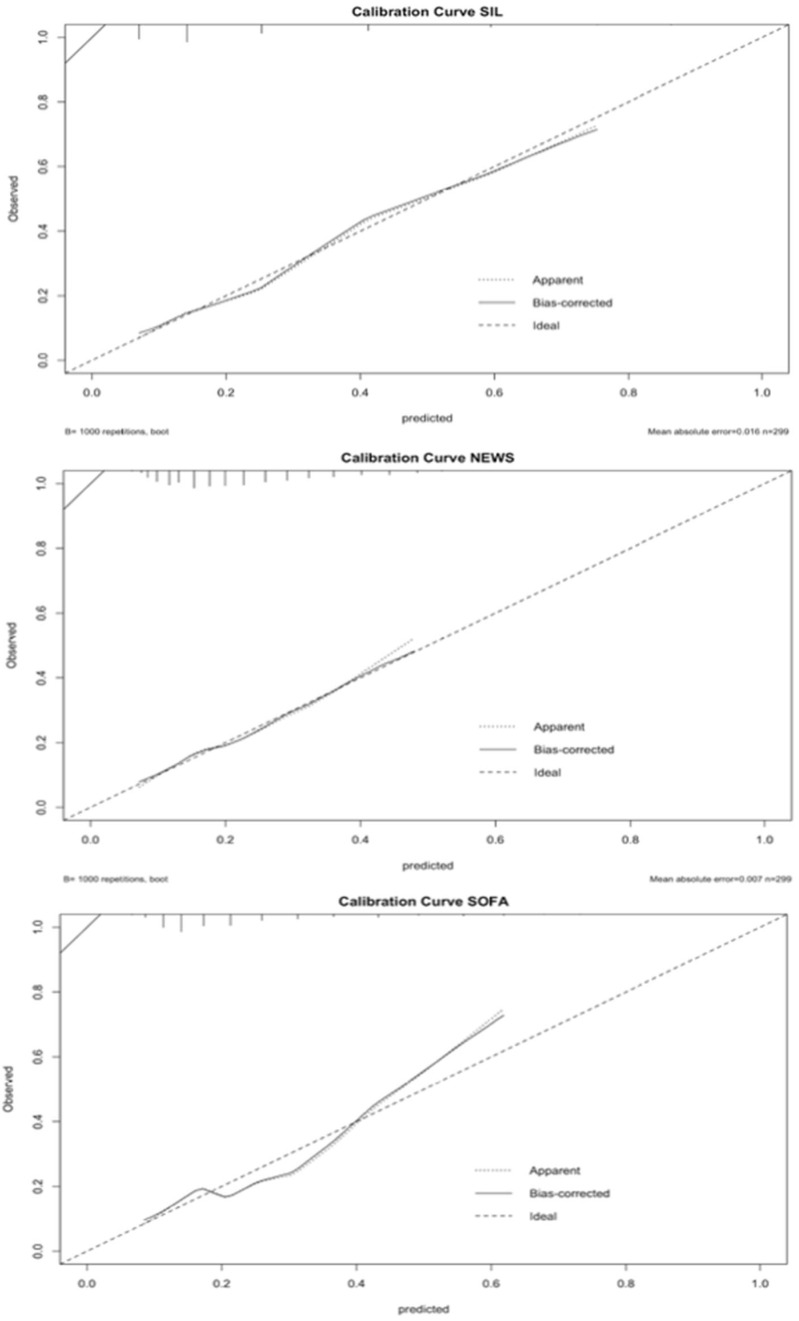
Calibration plots of the evaluated scales.

No differences in mortality were found based on the patients’ sex, whereas age, NEWS, qSOFA, SIL, and SOFA scores were shown to be significantly associated with mortality prediction (Table 2). Regarding the discriminatory ability of the analyzed tests, the SIL score exhibited the highest AUC at 0.754 (95% CI: 0.680–0.825) and *p*:0.01 (Figure 2). The AUCs of the other scales were as follows—SOFA: 0.663 (95% CI 0.578–0.750), NEWS: 0.655 (95% CI 0.573–0.7339), and qSOFA 0.352 (95% CI 0.280–0.425). We decided to exclude the qSOFA scale from the analysis due to its low discrimination capacity. Additionally, we made calibration curves for each scale (Figure 3).

The test with the highest sensitivity and specificity was the SIL score with 69.4 and 67.8%, respectively (Table 3). Additionally, the relationship between the expected mortality according to the SIL score value and that recorded in the study population was documented (Table 4).

## 4. Discussion

Sepsis is a potentially fatal condition [1] that requires tools for the early identification of patients at high risk of death from their initial assessment in emergency department triages. Currently, there is no specific scale designed for this purpose, and the recommendation is to use several clinical prediction tools [8]. This study compared the discriminatory ability of a new prediction scale with the most used scales in a cohort of patients from a single hospital center.

The SIL score is a scale that combines a purely clinical variable, the shock index (as an indicator of a patient’s hemodynamic status), with a paraclinical variable, the arterial lactate level (as an indicator of a patient’s metabolic status). This study showed that the area under the curve of the SIL score is comparable to those of the commonly used clinical scales, and it was found to even be superior to other clinical scales with an AUC of 0.754 (95% CI: 0.680–0.825). In this study, we found that the discrimination capacity for predicting the mortality of qSOFA is too low. For this reason, we did not use this scale for our comparative analyses. A cohort study involving 2045 patients demonstrated that qSOFA had a relatively low AUC of 0.63 (95% CI: 0.58–0.67) for mortality prediction [9]. Similarly, a study with 260 patients also exhibited a low predictive capacity for mortality with this score in an emergency department with an AUC of 0.605 (95%, CI: 0.503–0.706) [10]. Another cohort of 179 elderly patients demonstrated an even lower predictive capacity for death with an AUC of 0.559 (95%, CI: 0.485–0.663) [11]. In contrast, other studies have shown better predictive capacity, where the AUC for qSOFA mortality was 0.758 [12]. The meta-analysis by Wang et al. [13], which included 62,338 patients, demonstrated a slightly higher predictive capacity of qSOFA for mortality with an AUC of 0.69 (95% CI: 0.65–0.73) [13]. However, these data demonstrate that this scale alone is not sufficient as the sole tool for predicting the mortality in patients with suspected sepsis in an emergency department.

Additionally, when evaluating the performance of the NEWS, this study demonstrated a low predictive capacity for mortality with an AUC of 0.655 (95% CI: 0.573–0.733). These results are similar to those reported by Ruangsomboon et al. [14], whose study with a sample of 1622 patients showed an AUC of 0.61 (95% CI: 0.58, 0.64) [14]. A meta-analysis reported a slightly higher predictive capacity with an AUC of 0.69 (95% CI: 0.65–0.73) [13]. Based on these reports, the NEWS cannot be considered as a standalone tool for predicting mortality in patients.

Regarding the mortality prediction capacity of the SOFA score, interestingly, this study did not report a high discriminatory ability when used from the outset of patient care in an emergency department. This study demonstrated that the SOFA score has a low mortality prediction AUC of 0.663 (95% CI: 0.578–0.750), i.e., values that are comparable to the predictive capacity of other clinical scales that were investigated in this study. In a previous sample, it was reported that the mortality prediction capacity for sepsis in emergency departments in an elderly population is not very high, with an AUC of 0.662 (95% CI: 0.584–0.739) [11]. Similarly, a cohort with patients that had characteristics similar to this study demonstrated that SOFA has a low capacity for predicting death in emergency departments with an AUC of 0.63 (95% CI: 0.55–0.70) [15]. In contrast, in a previous publication where SOFA had slightly better mortality prediction capacity, with an AUC of 0.68 (95% CI: 0.64–0.73) [9], as well as according to a Korean study of 7393, the AUC for 28-day mortality was 0.75 (CI: 0.725–0.776) [16]. It is crucial to report that SOFA appears to have better prognostic performance when applied to populations entering intensive care with an AUC of 0.753 (99% CI: 0.750–0.757) for mortality [17]. This allows us to conclude that this scale is also not ideal as the sole tool for determining the severity and prognosis of patients with sepsis in emergency departments.

Regarding the other prediction measures, the sensitivity values of the scales used in this study demonstrated that the SIL score has a sensitivity of 69.4%, and this value was found to be the highest of all the scales analyzed. A study of a population of middle- and low-income individuals showed that the sensitivity of qSOFA for predicting mortality was 96% [15]. However, a recent meta-analysis demonstrated a sensitivity of only 46% [13], i.e., data that are similar to the sensitivity value reported in this study for qSOFA (38.3%). On the other hand, the SIL score proved to be the most specific scale with 67.8% when compared to the other scales evaluated in this study. Some studies have shown qSOFA specificities ranging from 0.82 (95% CI: 0.76–0.86) [13] and 42% [5], as well as NEWS specificities from 85% [5] to 0.52% (95% CI: 0.39–0.65) [12]. Therefore, the SIL score emerged as an intriguing tool based on the specificity and sensitivity values found.

Our results indicate that the predictive ability for the mortality of patients with suspected sepsis from the triage room of emergency departments remains low. Based on the AUC of the different scores, it seems preferable to use a scale that combines clinical and paraclinical variables, such as the SIL score, rather than solely clinical ones like qSOFA and NEWS. Additionally, these scales should be quickly applicable, making the SOFA score less useful at the outset of patient assessment since the paraclinical parameters it employs take longer to report than the lactate level used by the SIL score.

The predictive values of the SIL score, both negative (NPVs) and positive (PPVs), were 36.2% and 89.3%, respectively. This score exhibits a low NPV; however, similar results were observed with the other scales: 13.4% for qSOFA and 23.0% for NEWS. Interestingly, the SIL scale had a high positive predictive value of 85.9%, which was similar to the other scales evaluated (qSOFA at 70.4% and NEWS at 85.8%). However, with a positive predictive value of 89.3%, the SIL score effectively identified patients at high risk of mortality, allowing for more aggressive early intervention.

We evaluated the calibration performance of the SIL, NEWS, and SOFA scales. We performed a bootstrapping method with 1000 repetitions for each test and found that SIL and NEWS had a similar discrimination capacity, while the performance of SOFA was slightly lower, especially at extreme values. We used bootstrapping for the calibration curve because the data did not have a normal distribution, the sample was not that large, and because we considered that this method could mitigate some probable biases (Figure 3).

Regarding the relationship between the expected mortality behavior based on the SIL score value when compared to the value found in this study, there was a reasonable correlation with scores of 0 and even 3 points. However, with higher values of four and above, a good correlation was not found. Nevertheless, it is important to mention that this study demonstrated a linear relationship between the score value and the documented mortality; in other words, a higher score value corresponded to a higher mortality. A better correlation was not found for values of four or more due to the number of patients with elevated scores in this study.

This study reported an overall mortality rate in the cohort of 20.4%, which is comparable to other clinical studies showing overall sepsis mortalities of 24.9% [18] and 22.4% [19]. Regarding the comorbidities of the patients, we were only able to observe a significant association between having renal failure and mortality due to sepsis. It is likely that these patients had more alterations in the inflammatory response compared to the others; however, this condition is beyond the scope of this article. In terms of specific groups, the mortality rate for septic shock documented in this study was 39.6% of the total sample, which is comparable to findings in other studies such as the meta-analysis by Bauer et al., who showed a septic shock mortality rate of 34.7% [20], and the study by Fleischmann et al., where a mortality rate of 26% was reported [21]. Interestingly, the mortality due to sepsis in the studied cohort was low when compared with that reported in the literature, i.e., 9.8% vs. 24.4% in the meta-analysis by Bauer et al. [20] and the 17% reported in the study by Fleischmann et al. [21]. However, among the group of patients who died, 68.8% had septic shock, highlighting the importance of early recognition and prompt management of this condition.

### 4.1. Limitations

The main limitation of this study was related to the fact that the scales were evaluated in a single hospital center, which means that the results should be interpreted with caution when applying the scales to other populations. Additionally, the cohort of patients analyzed did not present many severely ill cases, which subtly limited the analysis of the behavior of the SIL score in this type of population. Despite being a single-center study, this research involved a well-characterized patient cohort, and the mortality rates observed were in line with those reported in the literature, supporting the reliability of the SIL score’s predictive capacity.

### 4.2. Implications

This study demonstrated that a scale that combines clinical and easily interpretable paraclinical variables in emergency departments, called the SIL score, is superior for predicting death from sepsis than the usual prediction scales that only use clinical variables. This tool could be considered in the triage areas of emergency departments to classify patients with a higher risk of mortality more objectively for the purpose of more timely care. Based on the results of this study, there is a need for a multicenter validation study of the SIL score method.

## 5. Conclusions

The SIL score is a useful bedside scale for predicting the in-hospital mortality in the triage area of emergency departments, and its discriminatory ability is superior to scales that only use clinical variables.

## Figures and Tables

**Table 1:** SIL score.

Shock Index *	Score (PIS)	Lactate (mmol/L)	Score (PL)
0–0.9	0	0–2.0	0
0.91–1.35	1	2.01–4.0	1
1.36–1.8	2	4.01–6.0	2
≥1.81	3	≥6.01	3
SIL score = (PIS + PL)			
	Minimum 0		
	Maximum 6		

* Shock index: heart rate/systolic pressure. PIS: shock index score range. PL: lactate score range. SIL: Shock Index/Lactate.

**Table 2:** Patient characteristics.

Variable	Survivors (%)	No. of Survivors (%)	OR	*p*
Total (*n* = 299)	238 (79.6)	61 (20.4)		
Sex (female)	145 (60.9)	30 (49.2)	1.60 (0.91–2.84)	0.130
Age (median (IQ))	72 (58–80)	74 (66–86)		0.03
Sepsis	174 (90.1)	19 (9.8)	0.16 (0.08–0.30)	<0.001
Septic shock	64 (60.3)	42 (39.6)	5.94 (3.25–11.21)	<0.001
qSOFA (median (IQ))	2 (1–2)	2 (2–3)		<0.001
NEWS (median (IQ))	8 (5–10)	10 (7–13)		<0.001
SOFA (median (IQ))	5 (4–6)	6 (4–10)		<0.001
SIL score (median (IQ))	2 (1–3)	3 (2–4)		<0.001
**Comorbidities** Arterial hypertension Mellitus diabetes Renal insufficiency Immunosuppression COPD	103 (78.0) 55 (77.4) 16 (53.3) 47 (78.3) 18 (64.2)	29 (21.9) 16 (22.5) 14 (46.6) 13 (21.6) 10 (37.1)	1.18 (0.67–2.09) 1.18 (0.60–2.23) 4.11 (1.84–9.08) 1.10 (0.53–2.17) 2.40 (1.00–5.46)	0.650 0.732 <0.001 0.926 0.062
**Sepsis origin (%)** Pulmonary (28.8) Urinary (20.7) Biliary (9.4) Abdominal (6.7) Soft tissues (5.0) Gastroenteritis (3.7)	66 (27.7) 55 (23.1) 22 (9.2) 15 (6.3) 11 (4.6) 9 (3.8)	20 (32.8) 7 (11.5) 6 (9.8) 5 (8.2) 4 (6.6) 2 (3.3)		0.832

qSOFA: Quick Sequential Organ Failure Assessment, NEWS: National Early Warning Score, SOFA: Sequential Organ Failure Assessment, SIL: Shock Index/Lactate, and COPD: chronic obstructive pulmonary disease.

**Table 3:** Comparison of the prediction tools’ sensitivity, specificity, and predictive values.

SCORE	SPEC	SENS	NPV	PPV
qSOFA	0.372	0.383	0.134	0.704
NEWS	0.595	0.628	0.290	0.858
SOFA	0.581	0.665	0.308	0.861
SIL	0.678	0.694	0.362	0.893

SPEC: specificity, SENS: sensitivity, NPV: negative predictive value, and PPV: positive predictive value.

**Table 4:** Relationship between the expected and found mortality according to the value of the SIL score.

SIL Value	Expected (%)	Found *n* (%)	Total *n* (%)
0	11.8	6(6.8)	87(29.1)
1	23.4	15(14.4)	104(34.8)
2	34.8	11(21.1)	52(17.4)
3	42.2	17(47.2)	36(12.0)
4	66.3	8(53.3)	15(5)
5	85.4	1(50)	2(0.7)
6	90.4	3(100)	3(1)

## Data Availability

The datasets used and/or analyzed during the current study are available from the corresponding authors on reasonable request.

## Abbreviations

qSOFA	Quick Sequential Organ Failure Assessment
NEWS	National Early Warning Score
SOFA	Sequential Organ Failure Assessment
SIL	Shock Index/Lactate
COPD	Chronic obstructive pulmonary disease
AUC	Receiver operating characteristic curve
NPV	Negative predictive value
PPV	Positive predictive value
